# The Precious Potential of the Sacred Tree *Chamaecyparis obtusa* (Siebold & Zucc.) Endl. as a Source of Secondary Metabolites with Broad Biological Applications

**DOI:** 10.3390/ijms25052723

**Published:** 2024-02-27

**Authors:** Karol Maksymilian Górski, Tomasz Kowalczyk, Laurent Picot, Patricia Rijo, Mansour Ghorbanpour, Przemysław Sitarek

**Affiliations:** 1 Institute of Chemistry, Faculty of Science and Technology, Jan Długosz University in Częstochowa, 42-200 Częstochowa, Poland; k.gorski@ujd.edu.pl; 2 Department of Molecular Biotechnology and Genetics, University of Lodz, 90-237 Lodz, Poland; 3 Littoral Environnement et Sociétés UMRi CNRS 7266 LIENSs, La Rochelle Université, 17042 La Rochelle, France; laurent.picot@univ-lr.fr; 4 CBIOS-Research Center for Biosciences & Health Technologies, Universidade Lusófona de Humanidades e Tecnologias, 1749-024 Lisbon, Portugal; patricia.rijo@ulusofona.pt; 5 iMed.ULisboa—Research Institute for Medicines, Faculdade de Farmácia da Universidade de Lisboa, Av. Prof. Gama Pinto, 1649-003 Lisbon, Portugal; 6 Department of Medicinal Plants, Faculty of Agriculture and Natural Resources, Arak University, Arak 38156-88349, Iran; m-ghorbanpour@araku.ac.ir; 7 Department of Biology and Pharmaceutical Botany, Medical University of Lodz, 90-151 Lodz, Poland; przemyslaw.sitarek@umed.lodz.pl

**Keywords:** *Chamaecyparis obtusa*, Hinoki, human-friendly insect repellent, biological activity, Japanese sacred tree, COEO

## Abstract

*Chamaecyparis obtusa* (Siebold & Zucc.) Endl., which belongs to the *Cupressaceae* family, occurs naturally in North America and Asia, especially in Korea, Taiwan and Japan, where it is an evergreen, coniferous, sacred, ethnic tree. It has many useful varieties that are widespread throughout the world and grown for decorative purposes. It is most commonly used as an ornamental plant in homes, gardens or parks. It is also widely used in many areas of the economy; for example, its wood is used in architecture as well as furniture production. In addition, oil extracted from *Chamaecyparis obtusa* is increasingly used in cosmetology for skin care. Due to its wide economic demand, mainly in Japan, it represents the largest area of plantation forest. Despite this, it is on the red list of endangered species. Its use in ethnopharmacology has led to more and more research in recent years in an attempt to elucidate the potential mechanisms of its various biological activities, such as antimicrobial, antioxidant, anticancer, antidiabetic, antiasthmatic, anti-inflammatory, antiallergic, analgesic and central nervous system effects. It has also been shown that *Chamaecyparis obtusa* can be used as an insect repellent and an ingredient in plant disease treatment. This thesis provides a comprehensive review of the biological studies to date, looking at different areas of the economic fields of potential use of *Chamaecyparis obtusa*.

## 1. Introduction

Cupressaceae is a family of coniferous plants dispersed worldwide. It includes more than 130 species divided into about 30 genera, including Thuja, Cupressus or Juniperus. The family includes many useful species used in various industries. Often the wood of some species is used in architecture, the furniture industry or the catering industry (in spices and alcohol production) [1,2]. In turn, essential oils extracted from this family are used in the cosmetic or perfume industry. Currently, one of the popular uses of species belonging to the Cupressaceae family is their cultivation as ornamental plants [2]. A wide range of secondary metabolites have been identified in this family, but terpene compounds, such as monoterpenes, diterpenes and sesquiterpenes, deserve special attention [3]. The rich content of secondary metabolites explains the use of some species in folk medicine. For example, *Juniperus drupacea* is used in Turkey as a remedy for abdominal pain, hemorrhoids, inflammation of the urinary tract, colds or diarrhea [4]. Furthermore, in America, *Juniperus scopulorum* was used by the Indigenous people to treat coughs, fevers and colds [5], while *Thuja koraiensis*, the leaves of which are rich in vitamin C, was used as a remedy for scurvy [6]. 

**Figure 1:**
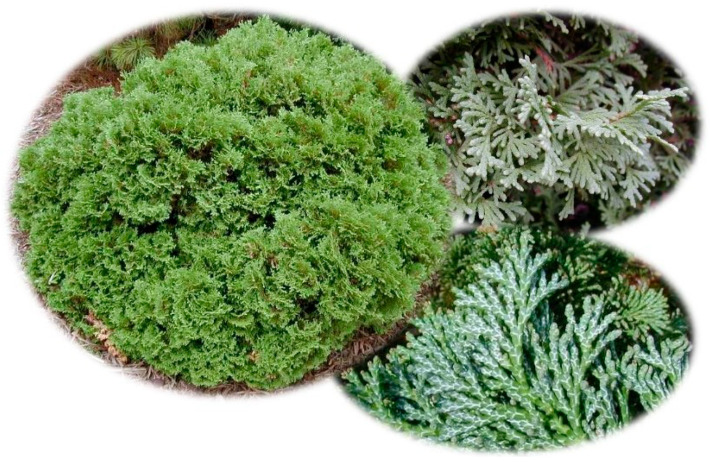
*Chamaecyparis* sp.

One of the more interesting species in the Cupressaceae family is *Chamaecyparis obtusa* (Siebold & Zucc.) Endl. (Figure 1), which is most commonly found in Asian countries, mainly in Japan, but also in Korea and Taiwan. It is an evergreen conifer with relatively slow growth, good tolerance to dry and arid soils and poor cold and salt tolerance, showing some resistance to air pollution [7]. Also known as Hinoki (meaning ‘fire tree’), it accounts for 25% of plantation forests in Japan. The current planting area of this species is the largest of all commercially cultivated forests in Japan [8]. Its wild form is one of the most visually prominent trees, reaching up to 35 m in height. In Taiwan, the most common wild variety is *Chamaecyparis obtusa* var. *formosana* [9]. *Chamaecyparis obtusa* (Siebold & Zucc.) is widely used in various industries. Currently, the plant is most commonly used for decoration. In addition, some dwarf varieties are grown as ornamental houseplants or bonsai trees. The most important varieties include ‘Crippsii’ and ‘Tetragona’ as well as the small and dwarf varieties ‘Nana’, ‘Nana Gracilis’, ‘Kosteri’, ‘Flabelliformis’, ‘Pygmaea’ and others [10]. It has been used in Japanese architecture for centuries, at least since the Nara era (710 AD), and the best example of this is the Horyuji Temple, one of Japan’s oldest wooden objects [11]. In Korea, on the other hand, it is used to make wooden fragrance cubes, moisturizers and cushions [12]. Currently, more and more studies are emerging, describing its medicinal and human health-enhancing properties. In this comprehensive review, we decided to collect articles describing the biological properties of *C. obtusa* in order to systematize and characterize the main directions of current biological research.

## 2. Phytochemistry of *Chamaecyparis obtusa*

**Figure 2:**
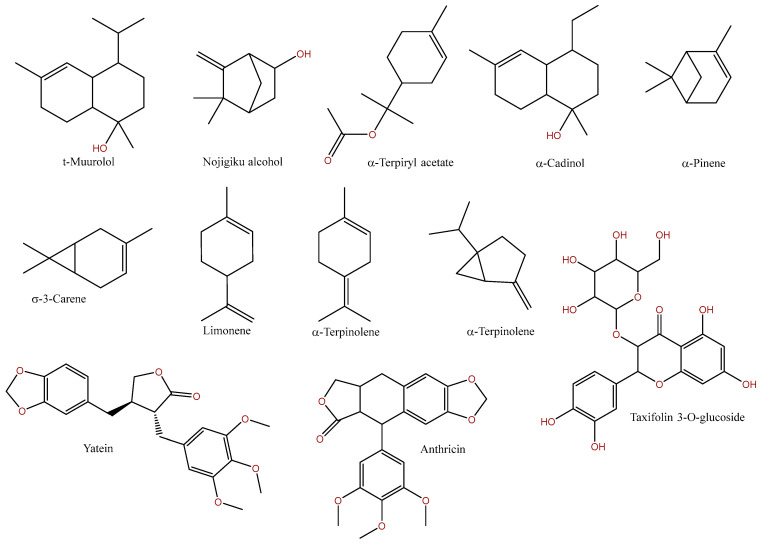
The most common compounds in *Chamaecyparis obtusa*.

*Chamaecyparis obtusa* is rich in a large variety of secondary metabolites, which have been identified in different plant organs. Groups of compounds, such as flavonoids, sterols, alkaloids, triterpenes, phenols, cardiac glycosides and tannins, have been found in the ethanol extract of the fruit [13]. Flavonoids are secondary metabolites of the polyphenol group, and their overall structure contains two phenolic rings and one heterocyclic ring with an oxygen atom. In plants, they have various functions, such as pigments, growth regulation and antioxidants. Flavones (quercetin, myricetin and amentoflavone) [14] and the biflavones bilobetin, sciadopitin, ginkgetin, podocarpusflavone B, isoginkgetin and 7,7″-O-dimethylamentoflavone, among others, are present in leaves. Hinokiflavone, podocarpusflavone A, 7-O-methylamentoflavone and amentoflavone represent chemotaxonomic signatures of the genus *Chamaecyparis* [15]. In addition, other compounds of the flavonoid group also were obtained from the leaves, including glucodistilin (Taxifolin 3-O-glucoside) and its isomers [16], ginkgetin and amentoflavone, which belong to the biflavonoid group, deoxypodophyllotoxin (anthricin), which belongs to the furonaphthodioxoles [17,18]. Another group includes lignans belonging to the class of polyphenols, where the leading structure is two bound phenylpropanoid units and the bonding occurs between the beta positions in the propane side chains. The role of this class of compounds is not yet well understood, but they are thought to be essential defensive mechanisms in plants. Lignans such as hinokinin, norlignan and hinokiresinol have been isolated from the heartwood [19] and yatein and 9-O-(11-hydroxyeudesman-4-yl)-dihydrosesamine from *Chamaecyparis* sp. leaves [20,21]. Terpenes are a large class of secondary metabolites that can have many functions. Primarily, they are responsible for the plant’s odor, which can be used to attract insects but also to repel them. In addition, high concentrations of terpenes can be toxic, which provides a certain protective function for the plant against herbivores. In general, the formula of terpenes is (C_5_H_8_)_n_, where n specifies the isoprene subunit count. Depending on the size of n, and thus the size of the molecule, terpenes can be divided into several groups, the most important being triterpenes, diterpenes, sesquiterpenes and monoterpenes [22]. The presence of 14 mono-, four sesquiterpenes and 10 diterpenes, as noted in a-terpinyl acetate, terpinen-4-ol, trans-sabinene hydrate, longifolene, thujopsene, ferruginol and hinokione, have been identified in the seeds of this species [23]. Additionally, quinonomethidic compounds, such as chamaecidin, isochamaecidin and chamaecidinol, were isolated from the seeds [24]. In contrast, Shieh et al. reported the presence of 62 different mono- and sesquiterpenes in essential oils from different parts of *Chamaecyparis obtusa* [25]. In addition, Fukushima et al. isolated abietane- and labdane-type diterpenoids and a triterpene from the cones [26]. Studies show that the composition of essential oils is significantly influenced not only by the part of the plant, but also by its place of origin. Fujise et al. compared oil obtained from the leaves and wood of *C. obtusa* and indicated differences from commercial oil [27]. Another class of compounds, similar to terpenes, are the terpenoids, where the ‘isoprene rule’ is not required to be followed and the compounds may have other functional groups [28]. An example of such a compound, isolated from *C. obtusa*, is β-thujaplicin (hinokitiol). In addition, a variety from Taiwan (*Chamaecyparis obtusa* var. *formosana*) showed the presence of new lignans, diterpenes and sesquiterpenes, such as muurola-4,10(14)-dien-3-one, 10-O-acetyl-15-oxo-R-cadinol, 4R-hydroxy-5β-ethoxy-epi-cubenol, obtusanal B, obtusadione, chamalignolide and chamaecyformosanin [29,30,31,32]. Phytochemical studies to date indicate that the most significant raw material extracted from *Chamaecyparis obtusa* appears to be the essential oil. This is indirectly confirmed by biological studies, most of which are specifically concerned with the essential oil and today’s areas of use (the production of cosmetics or shampoos). Essential oils are the volatile fraction of secondary metabolites of plants, usually obtained by steam distillation, and are characterized by a strong fragrance. In addition to terpenoids and terpenes, aliphatic components and phenol-derived aromatic components are also present in essential oils. Due to their rich content of various chemical compounds, essential oils show a large spectrum of bioactivities, such as antibacterial, analgesic, anti-inflammatory, antioxidant, and anticancer [33,34]. Of the 3000 known essential oils, 300 are an important raw materials for agricultural, food, perfume, sanitary, cosmetology and pharmaceutical industries [35]. *C. obtusa* essential oil from various parts of this plant has been shown to contain t-muurolol, nojigiku alcohol, α-terpinyl acetate or α-cadinol [25]. In other studies of essential oil from branches of the plant, the main constituents were δ-cadinene and α-pinene [36], while the main compounds in the oil from the fruit were α-pinene and δ-3-carene [37]. There may be differences between the different essential oils. For example, in leaf oil, (+)-limonene was most commonly detected [38], while in other studies the main compounds were α-terpinolene, (+)-3-carene, α-pinene [39] or α-terpinyl acetate, sabinene and isobornyl acetate [40]. As mentioned earlier, these differences may be due to the place of origin of the plant, but also to the way the oil was distilled. Other important compounds obtained from the essential oil of *C. obtusa* are kaur-16-ene, nezukol, cuminol, eucarvone and calamenene [41,42]. Selected chemical formulas of the most common compounds in this species are shown in Figure 2.

## 3. Antimicrobial and Antivirus Activity

The growing problem of superbugs and antibiotic resistance is forcing scientists to search for new antibiotics, and plants can be a source of valuable new compounds. The essential oil of *Chamaecyparis obtusa* (COEO) shows numerous beneficial biological properties, some of the most important being precisely the antimicrobial and antifungal properties. Kim et al. showed that *Chamaecyparis obtusa* leaf extract is effective against one of today’s most characteristic bacteria with the ability to resist antibiotics, methicillin-resistant *Staphylococcus aureus* (MRSA). The authors showed that the resulting essential oil in concentrations greater than 0.1 mg/mL inhibited MRSA growth, acid production from glucose metabolism and biofilm formation. Furthermore, it inhibited the virulence factor genes agrA and sea as well as sarA at concentrations greater than 0.2 mg/mL and 0.3 mg/mL, respectively [43]. The fresh leaf extract not only shows strong antibacterial activity against Gram (+) bacteria, such as *S. mutans*, *S. pyogenes*, *L. monocytogenes*, *L. mesenteroides*, *L. plantarum* and *B. cereus*, but also has antifungal activity against *P. ovale* and *S. cerevisiae*, as shown by Yang et al. [40]. The antimicrobial efficacy of the broad-spectrum essential oil may be due to a mechanism affecting the microbial cell membrane integrity. This is supported by a study by Bajpai et al., who investigated the minimum bactericidal concentration (MBC) and minimum inhibitory concentration (MIC) of essential oil extracted from *Chamaecyparis obtusa* against foodborne pathogens, such as *B. cereus*, *L. monocytogenes*, *S. aureus*, *E. coli* and *S. typhimurium*. In addition, the authors showed that COEO causes cell membrane rupture or significant morphological changes in *B. cereus* and *E. coli.* Moreover, efflux of potassium ions and a release of extracellular adenosine 5′-triphosphate (ATP) and cellular material were demonstrated in these bacteria, confirming the effect of the COEO on the integrity of cell membranes of both Gram-negative and Gram-positive bacteria [44]. Inhibition of the growth of certain bacterial strains was also demonstrated by Park et al. The fraction that included terpinen-4-ol (TF) showed the most significant inhibitory effect against all strains tested. In addition, this compound was tested in vivo in a mouse model of *S. aureus* infection. A histopathological examination showed that TF reduced the inflammation caused by this bacterium [45]. Another antibacterial compound obtained from *C. obtusa* is β-thujaplicin (hinokitiol). Koyama et al. demonstrated the antibiotic effect of the essential oil antibiotics and β-thujaplicin on MRSA. In their study, they further showed that another compound, Yoshixol, was mainly responsible for the antibacterial effect of essential oils [46]. In another study, three compounds (chamaecyformosanin A, chamaecyformosanin C and chamaecyformosanin D) isolated from the bark of a variety of this species found in Taiwan (*C. obtusa* var. *formosana*) showed antimicrobial activity against molds, including *S. cerevisiae*, *P. italicum*, *A. niger* and *C. albicans*, and numerous bacterial strains, for example *P. aeruginosa*, *E. coli* and *S. aureus* [31]. Other studies also confirm the antibacterial and antifungal properties of *C. obtusa* [47]. In another study, an antifungal effect against *M. furfur* reducing dandruff was observed in individuals after application of *C. obtusa* essential oil [48]. In contrast, the use of essential oils in dental preparations inhibits the growth of *S. mutans*, which is one of the main microbial reasons for tooth decay and biofilm (plaque) formation. COEO reduced acid production and the formation of microbial biofilm and suppressed the expression of various virulence factors genes [49]. Not only the essential oil, but also the non-volatile residues show inhibitory activity against drug-resistant strains of clinical origin, including infectious bacteria and methicillin-resistant strains of *S. aureus* and vancomycin-resistant *enterococci* (VRE) [50]. In addition to the described antibacterial and antifungal activities, *C. obtusa* has antiviral activity. Kuo et al. isolated the compound yatein from a methanolic extract of dried *C. obtusa* leaves, which inhibited HSV-1 replication in HeLa cells without any cytotoxic effects. Yatein also decreased ICP0 and ICP4 gene expression and impaired the formation of the α-trans-induction factor/C1/Oct 1/GARAT multiprotein complex [21].

### 3.1. Antioxidant Activity

Oxidation reactions are an essential part a wide variety of cells processes and, although crucial for life, can be harmful. They produce free radicals that can cause oxidative stress in the cell, which has a fundamental impact on the aging process and the course of many diseases, such as atherosclerosis, cancer, chronic kidney failure, diabetes, neurological disorders, Alzheimer’s and Parkinson’s dementia and other inflammatory diseases. Antioxidants, on the other hand, are molecules that protect against cell damage induced by free radicals. New antioxidants are currently being sought not only as dietary supplements, but also as drug candidates that could be used to treat or prevent diseases, in the development of which free radicals play an important role [51,52]. Studies on *Chamaecyparis obtusa* also detect its antioxidant activity [53]. Eltayeb et al. demonstrated that the essential oil isolated from the fruit of *Chamaecyparis obtusa* showed anti-ABTS activity and total antioxidant activity in a phosphomolybdenum assay [37]. The endemic variety, i.e., *Chamaecyparis obtusa* var. *formosana*, showed an antioxidant effect in the DPPH assay [54], which was confirmed in both in vitro and in vivo studies using the nematode *Caenorhabditis elegans* by Cheng et al. The authors also showed a reduction in lipofuscin (ageing pigment) deposition that simultaneously prolonged the life of *C. elegans* [55]. A similar effect was also shown for the essential oil obtained from dried sawdust, which inhibited DPPH, nitric oxide, superoxide and hydroxyl radicals, as well as ferric iron ion-induced lipid peroxidation [56]. In conclusion, the above studies confirm that *Chamaecyparis obtusa* can be used as a rich source of antioxidants with promising biological properties, as part of a healthy diet to delay ageing processes and as a natural way to prevent food spoilage.

### 3.2. Anticancer Activity

**Figure 3:**
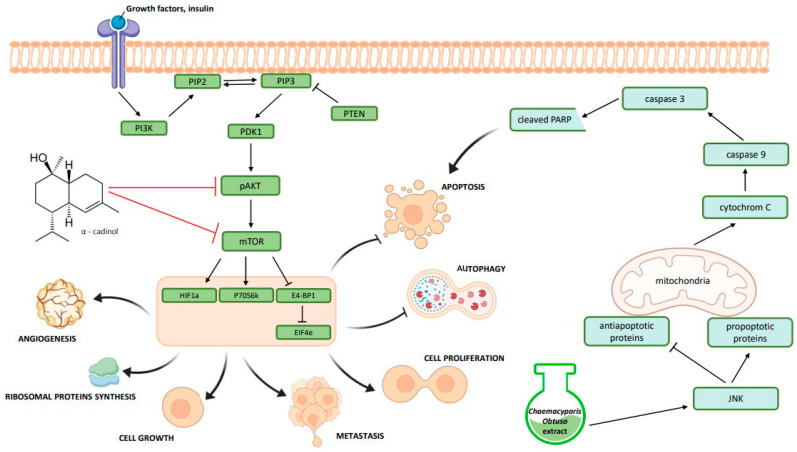
Potential anticancer mechanisms of action of *Chamaecyparis obtusa* extract and the isolated compound α-cadinol (created by BioRender).

In well-developed countries, the most common causes of death include cardiovascular disease and cancer, with the latter being one of the most serious diseases due to its course and therapeutic problems. According to cancer incidence estimates, 20 million new diagnoses were made, and 10 million people died from the disease [57]. Many of the medicines used at present have been discovered in nature or obtained by modifying natural leading compounds [58]. Many plants have been tested for potential cytotoxic properties, using not only extracts but also isolated, pure compounds [59]. Research on *Chamaecyparis obtusa* also confirms its anticancer properties. Dibwe et al. showed that the extract from this species is cytotoxic to human pancreatic cancer cells (PANC-1). A similar effect was observed for pure compounds, among which the most active one was α-cadinol, showing cytotoxicity to five human pancreatic cancer cell lines. Moreover, α-cadinol treatment resulted in inhibition of p-Akt expression and mTOR phosphorylation as well as excessive activation of autophagy in PNAC-1 cells [60]. Another study showed that aqueous extracts of *Chamaecyparis obtusa* leaves and the compound isolated from them, including anthricin, have cytotoxic effects against a colorectal cancer cell line (HCT116), but not against Chang’s liver cells. The authors demonstrated a mechanism of apoptosis induction through activation of the JNK pathway and expression of proteins including caspase-3, PARP, JNK, p-JNK, ERK and p38 [18]. Another compound showing promising cytotoxic activity is chamaecypanone C, which was isolated from *Chamaecyparis obtusa* var. *formosana* and reduced the survival of cell lines of various cancers, such as human oral epidermoid carcinoma (KB), human nasopharyngeal carcinoma (HONE-1) and human gastric carcinoma (TSGH) [61]. Moreover, the compound Yoshixol, which is found in the wood oil of *Chamaecyparis obtusa*, caused (like wood oil) a reduction in the proliferation of HeLa cancer cells and also induced DNA fragmentation [62]. In other studies, Kwon et al. showed that *Chamaecyparis obtusa* leaf extract (CO99EL) inhibited the activation of the STAT3 transcription factor, which regulates the expression of various oncogenes in cell lines of many different cancers. As a result of further experiments, the authors observed that this extract inhibited cell migration in MDA-MB-231 and reduced the levels of epithelial-mesenchymal transition (EMT) marker proteins, such as N-cadherin, fibronectin and TWIST, and the levels of CDH2, FN1, TWIST1, MMP2 and MMP9 genes, which suggested that it could inhibit breast cancer metastasis by suppressing various EMT-related genes. Furthermore, this extract also induced apoptosis in the same MDA-MB-231 line by reducing the levels of apoptosis-inhibiting proteins, including Bcl-2 and Bcl-xL. In this study, the authors also demonstrated the in vivo effect in a mouse model of breast cancer after treatment with *Chamaecyparis obtusa* extract, which showed tumor reduction and inhibition of metastasis to other organs [63]. The potential mechanism of anticancer activity based on described studies is shown in Figure 3. 

### 3.3. Antidiabetic Activity

**Figure 4:**
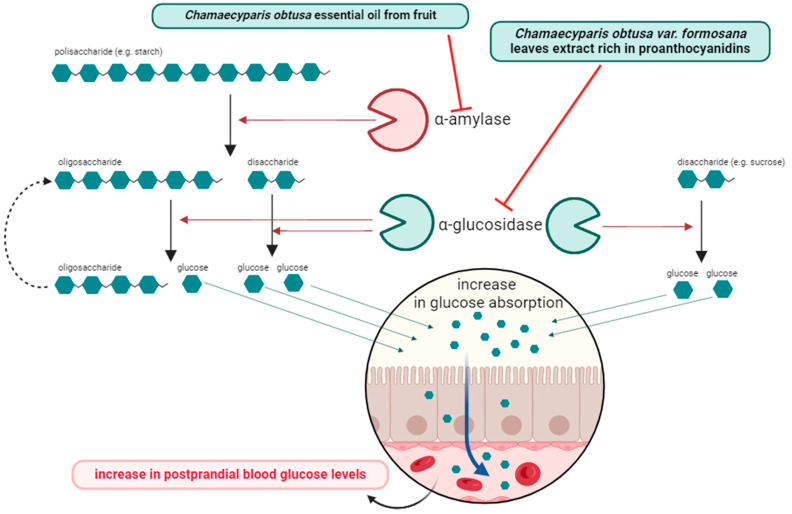
Potential antidiabetic mechanism of *Chamaecyparis obtusa* fruit essential oil and *Chamaecyparis obtusa* var. *formosana* extract (created by BioRender).

Diabetes is a non-infectious illness that definitely poses a growing problem in modern society. According to the WHO data, in 2014, the population of diabetics was 422 million. In addition, it was estimated that in 2019, the number of deaths directly caused by the disease was 1.5 million, while 2.2 million people died from hyperglycemia. The development of diabetes is complex and primarily involves increased blood glucose concentrations and impaired insulin action or production, resulting in insulin resistance [64]. In low- and middle-income countries, a very rapid increase in its prevalence has been observed. The search for compounds of natural origin may not only increase the availability of antidiabetic therapy in areas where drug availability is limited, but may also allow new therapeutic objectives to be found. Hence, natural ways of treating diabetes appear to be very relevant [65,66]. One of the mechanisms of how antidiabetic drugs work is to block alpha-amylase, an enzyme that hydrolyses large sugar molecules, such as starch, into smaller molecules, which increases blood glucose concentrations as a result [67]. Eltayeb et al. showed that essential oil extracted from *Chamaecyparis obtusa* fruit inhibits alpha-amylase activity in an in vitro study [37]. Another therapeutic approach for the treatment of diabetes is the inhibition of alpha-glucosidase, which leads to the inhibition of the breakdown of alpha-bound carbohydrates, reducing the absorption of glucose into the blood from the gastrointestinal tract and resulting in a reduction in postprandial glycemia. This effect has been demonstrated using an aqueous extract of *Chamaecyparis obtusa* var. *formosana* leaves rich in proanthocyanidins [68]. Antidiabetic properties were also confirmed in vivo in rats with streptozotocin-induced hyperglycemia and a high-fat diet. After three months of treatment, both groups of rats demonstrated improvements in glucose metabolism in oral glucose tolerance tests and post-meal blood glucose tests. In addition, the extract tested caused a reduction in HOMA-IR, leptin and adiponectin levels [69]. These activities indicate that *Chamaecyparis obtusa* (and especially var. *formosana*) can be used in the complementary treatment of diabetes and provide a starting point for the search for new antidiabetic compounds. The proposed antidiabetic mechanism is shown in Figure 4.

### 3.4. Antiasthmatic Activity

Asthma is a disease associated with inflammation and a chronic course. Immune cells have been demonstrated to take an important part in the pathogenesis of this disease by inducing bronchial hyperresponsiveness, leading to overproduction of mucus and airway constriction [70]. It is estimated that up to 300 million people worldwide suffer from asthma, regardless of a country’s level of development. The cost of treating asthma cases in the US exceeds $18 million per year [70,71]. In addition to high costs, current asthma therapy is characterized by side effects that reduce patients’ adherence to the prescribed treatment. In this case, the use of natural products could not only reduce the cost of treatment, which would certainly increase access to therapy for people from all socio-economic backgrounds, but could also increase their adherence to the treatment plan due to reduced side effects [71]. The literature reports that *Chamaecyparis obtusa* could be one of the potential antiasthmatic drugs. A study conducted on an essential oil extracted from its leaves in an in vivo mouse model of ovalbumin-induced asthma showed a reduction in airway hyperresponsiveness, a reduction in Th2 cytokine levels and eosinophil counts in bronchoalveolar lavage fluid (BALF) and a reduction in serum levels of specific anti-OVA IgE. Terpenoid compounds were responsible for the biological effect and accounted for over 80% of the oil [72]. The effect of terpenes on alleviating asthma was also confirmed by Ahn et al., who demonstrated the antiasthmatic effect of volatile organic compounds (VOCs) released from wooden panels (including panels made of *Chamaecyparis obtusa*) also using a mouse model of ovalbumin-induced asthma. Mice were housed in polyacrylamide chambers containing wooden panels from various conifers (including *Chamaecyparis obtusa*). The results indicated a reduction in granulocyte count and increased expression of cytokines TNF-α, IL-4, IL-9 and IL-13 compared to mice from the control group [73]. Due to the limited number of studies in this area, the above examples show that *Chamaecyparis obtusa* can be effectively used to alleviate asthma, although further research is needed, including clinical research, especially focusing on single, pure compounds isolated from this species.

### 3.5. Other Airway Diseases

In addition to its antiasthmatic effects, *Chamaecyparis obtusa* may be a potential agent for the treatment of other respiratory diseases, with a particular focus on inflammatory diseases. A phytoncide compound isolated from the *Chamaecyparis obtusa* essential oil reduced inflammation in WI38 fibroblasts by inhibiting LPS-induced activation of cyclooxygnase-2 and nitric oxide synthase and the degradation of NF-κB-α inhibitor and p65. In addition, there was an increase in alveolar capacity in rats subject to olfactory stimulation with the oil [74]. Additionally, volatile organic compounds (VOCs) of *Chamaecyparis obtusa* caused a reduction in the expression of various LPS-induced cytokines in cells obtained via bronchoalveolar lavage as well as from lung tissues and blood [75]. In contrast, microencapsulated *Chamaecyparis obtusa* essential oil inhibited the expression of various inflammatory mediators induced not only by LPS, but also by the fungus *Alternaria alternata* in monocyte-derived dendritic cells [76].

### 3.6. Dermatological Effects

Skin diseases are a serious problem that affects patients all over the world. The balance of this largest organ in our body is extremely important. For centuries, people have used plants for skin care and treatment of skin diseases. Recently, more and more research has focused on studying natural compounds for their potential use in healing skin diseases, such as melanoma, acne or atopic dermatitis, but also explaining the mechanism of action of plant compounds already used in clinical practice [77]. Atopic dermatitis (AD) is a serious inflammatory skin disease of a chronic or recurrent nature. It affects approximately 3% of the adult population and 20% of children [78]. The pathogenesis of AD is not fully understood. It has been shown that the pathogenic mechanisms are influenced by genetic, environmental, pharmacological and even psychological factors. The course of atopic dermatitis is characterized by eczematous skin lesions with excessive inflammatory cell infiltration of lymphocytes, macrophages and granular mast cells [79]. The *Chamaecyparis obtusa* essential oil has been shown to inhibit DNCB-induced AD-like lesions in BALB/c mice through inhibition of Th1/Th2 cytokine overexpression and IgE in both lymph node and spleen [80]. In addition, in NC/Nga mice, treatment with a fabric saturated with the essential oil produced a significant decrease in the SCORAD score with modifications in transepidermal water loss and serum IgE levels, a reduction in epidermal proliferation and changes in filaggrin, involucrin, and loricrin expression [81]. The antiatopic effect was also confirmed by Yang et al., who showed that volatile organic compounds from *Chamaecyparis obtusa* cause inhibition of pathological dermal changes, decrease mast cell infiltration, reductions in serum IgE levels and inhibition of IL-6 and IL-1β in a dose-dependent manner [82]. An important compound derived from *Chamaecyparis obtusa* in the treatment of atopic dermatitis (AD) is elemol, which decreased IgE serum levels, infiltration of mast cells into the hypodermis and dermis, and expression of TNF-α, IκBα, IL-6 and IL-1β in the skin of the 2,4-dinitrochlorobenzene-induced animal models of atopic dermatitis. Furthermore, mast cell lines (RBL-2H3) treated with both essential oils extracted from the wood of *Chamaecyparis obtusa* and elemol showed a reduction in IL-13 and IL-4 mRNA levels and β-hexosaminidase release from mast cells [83,84]. The above data suggest that *Chamaecyparis obtusa* can be used to reduce symptoms associated with atopic dermatitis (AD), as well as for the production of functional clothing supporting the treatment of atopic dermatitis therapy. 

Another dermatological problem that affects social interactions and the mental well-being of patients is alopecia [85]. Due to its antibacterial and antifungal properties, *Chamaecyparis obtusa* essential oil is used in various cosmetics, including shampoos that support hair growth [42]. This activity was confirmed in vivo in shaved mice, where the essential oil promoted early growth. Moreover, in vitro tests using the HaCaT cell line (human keratinocytes) indicated that certain oil sub-fractions induced an increase in the expression of vascular endothelial growth factor (VEGF) and keratinocyte growth factor (KGF) without up-regulating the hair growth inhibition factor, transforming growth factor β1 (TGFβ1). The compounds common to the sub-fractions showing this effect were cuminol, eucarvone and calamenene [42]. Another study showed that *Chamaecyparis obtusa* oil stimulated the activation of hair growth-related factors, such as alkaline phosphatase (ALP) and γ-glutamyl transpeptidase (γ-GT), in C57BL/6 mice. This may have contributed to higher blood flow, which supplies hair with the nutrients it needs for growth. In addition, the oil enhanced hair follicle cell proliferation and expression of hair growth factors (IGF-1 and VEGF) in mice [86]. The previously mentioned antioxidant properties of *Chamaecyparis obtusa* in a dermatological context may suggest its use in antiaging preparations. This is indirectly confirmed by a study by Jang et al., which showed that an ethanolic extract of *Chamaecyparis obtusa* leaves not only exhibits antioxidant activity, but also reduces UVA-induced fibroblast death. In addition, it increases the mRNA expression of superoxide dismutase and type I collagen, suggesting an antiwrinkle activity [87]. Moreover, *Chamaecyparis obtusa* bark extract also showed the effect of inhibiting melanogenesis. In alpha-melanocyte-stimulating hormone (α-MSH)-induced B16F10 murine melanoma cells, the extract caused inhibition of the expression of microphthalmia-associated transcription factor (MITF) and tyrosinase-related proteins (TRP1 and TRP2) and cAMP response element-binding protein (CREB) activation by suppressing AKT and extracellular signal-regulated kinase phosphorylation [88]. Also worth highlighting is the significant antiacne effect of *Chamaecyparis obtusa*, which was confirmed in a double-blind split-face clinical trial involving 34 patients. The study compared Lactobacillus-fermented *Chamaecyparis obtusa* (LFCO) and tea tree oil (TTO). After 8 weeks of treatment with LFCO, inflammatory lesions were reduced by 65.3%, while in the patients treated with the TTO, the lesions were only reduced by 38.2%. In addition, LFCO reduced the size of sebaceous glands and sebum output, and an improvement in non-inflammatory lesions by 52.6% was observed [89]. The studies described here point to the potentially very wide application of *Chamaecyparis obtusa* not only in the natural treatment of certain dermatological diseases, but also in the production of antiaging cosmetics, as well as in cosmetics promoting hair growth or reducing skin pigmentation changes. Furthermore, the fact that the lipid fraction of *Chamaecyparis obtusa* accelerates wound healing [90], combined with its antimicrobial activity, suggests that *Chamaecyparis obtusa* is a candidate for the treatment of injured skin.

### 3.7. Anti-Inflammatory Activities 

Inflammation is characterized as the immune system’s reaction to a variety of factors, including xenobiotics, pathogens, cellular damage or oxidative stress [91]. It is the organism’s way to restore cellular dysfunction caused by any damaging conditions and is often closely associated with the secretion of pro-inflammatory mediators, such as chemokines and cytokines (e.g., IL-6, IL-1 and TNF-a) [92]. Chronic inflammation can also cause negative effects in various tissues and can lead to the risk of developing a number of disorders, such as allergies, atopic dermatitis, atherosclerosis, asthma, diabetes, CNS diseases or cancer [91]. The search for new substances (also of natural origin) of an anti-inflammatory nature may contribute to broadening the prevention or therapeutic options for various chronic diseases. In vivo studies on rats after treatment with *Chamaecyparis obtusa* oil showed that it inhibits LPS-induced production of prostaglandin PGE2 and reduces the expression of transforming growth factor α (TNFα) and cyclooxygenase-2 (COX-2) [93]. In addition, it inhibits the production of compounds related to inflammation (nitric oxide, TNF-α, IL-6, nitric oxide synthase and COX-2) in LPS-stimulated RAW 264.7 cells. On the other hand, the oil reduced inflammation in two murine models of inflammation: carrageenan-induced peacock edema (the oil reduced peacock skin thickness and expression of IL-6 and IL-1β) and thioglycollate-induced peritonitis (TNF-α, IL-1β and IL-6 levels were reduced in peritoneal fluid) [94]. Additionally, *Chamaecyparis obtusa* leaf extract counteracted LPS-induced macrophage activation by inhibiting JAK/STAT in RAW264.7 cells and reduced the expression of many inflammatory mediators [95]. Due to the fact that terpenoids are compounds with anti-inflammatory properties, in studies comparing extracts obtained from different parts of *Chamaecyparis obtusa* (leaves, cones, wood and bark), the wood extract showed the strongest inhibition of nitric oxide (NO) production, and the degree of NO inhibition was proportional to the concentration of α-pinene [96]. Another compound isolated from *Chamaecyparis obtusa* with enormous anti-inflammatory potential is β-thujaplicin, which, both in vitro and in vivo, suppressed inflammation development more effectively than indomethacin, which is a non-steroidal anti-inflammatory drug (NSAID). Moreover, β-thujaplicin caused a significant reduction in mortality in mice suffering from septic shock [97]; therefore, *Chamaecyparis obtusa* may be an extremely interesting source of potential new anti-inflammatory drugs. A similar effect was observed for the oil obtained from *Chamaecyparis obtusa* var. *formosana* that inhibited the expression of various inflammatory mediators in murine macrophage cells, additionally inhibiting reactive oxygen species (ROS) and cellular signaling by affecting ERKI/2, JNKI/2 and p38 [98].

### 3.8. Antiallergic Effects

Inflammation is an integral part of the development of allergic diseases, and Th2 chemoattractants and cytokines are not only important in the pathogenesis of allergy, but are also responsible for most of the pathophysiological symptoms of allergy [99]. Anti-inflammatory drugs, e.g., glucocorticosteroids [100], and biological drugs targeting selected cytokines, e.g., reslizumab and mepolizumab targeting interleukin (IL)-5 [101], are used to treat this disease and other related diseases, such as asthma. The anti-inflammatory effect of *Chamaecyparis obtusa* oil may confirm its use in allergic conditions, as shown by some studies. For example, intranasal administration of the oil not only inhibited the expression of Th2 cytokines (IL-10, IL-4 and TNF-α) in nasal lavage fluid and activated splenocytes, but also reduced numerous nasal symptoms of an ovalbumin-induced mouse model [102]. In addition, some fractions of the oil suppressed the β-hexosaminidase secretion, which is characteristic of the development of an allergic reaction, and inhibited calcium influx, reactive oxygen species (ROS), and phosphorylation of ERK in rat basophilic leukemia cell line (RBL2H3) [103]. Furthermore, the oil reduced cytokine production in *Dermatophagoides pteronyssinus* and *Dermatophagoides farina* and stimulated human primary nasal epithelial cells (HNECs) [104].

### 3.9. Analgesic Effects

Inhibition of an inflammatory response may also contribute to pain relief. The essential oil from *Chamaecyparis obtusa* has been shown to have an antinociceptive effect in vivo by inhibiting peripheral pain perception pathways, which is associated with inhibition of various inflammatory mediators, including COX-2 [105]. A similar result was obtained in rats with carrageenan-induced arthritis, in which the essential oil improved the animals’ behavior in a voluntary walking test and reduced inflammation [106]. 

### 3.10. Activity in the Central Nervous System 

Some fractions or pure compounds obtained from *Chamaecyparis obtusa* may have an effect on the central nervous system (CNS). Examples include substances isolated from the leaves of *Chamaecyparis obtusa*, i.e., amentoflavone, ginkgetin and (-)-epitaxifolin 3-O-β-D xylopyranoside, which exhibited the effect of neuroprotection of murine hippocampal cells (HT22) against glutamate-induced oxidative damage. This effect was achieved by the strengthening of the activities of antioxidant enzymes, such as glutathione reductase (GR), superoxide dismutase (SOD), and/or inhibiting ERK1/2 activation [17]. Other compounds isolated from *Chamaecyparis obtusa* leaves, including hinokinin and 9-O-(11-hydroxyeudesman-4-yl)-dihydrosesamine, promoted PC12 cell growth in the neurite outgrowth activity test [20]. Additionally, leaf essential oil and heartwood extract have been shown to inhibit acetylcholinesterase activity. Kaur-16-ene, nezukol and ferruginol in the oil and (-)-hinokinin in the heartwood are responsible for this action. In turn, nezucol and ferruginol showed antibutyrylcholinesterase activity [41]. This suggests that *Chamaecyparis obtusa* may be a source of substances in the treatment of Alzheimer’s disease and other neurodegenerative diseases. The effect of *Chamaecyparis obtusa* in the CNS was also confirmed in in vivo studies. Studies have shown that inhalation of *Chamaecyparis obtusa* essential oil inhibits cognitive impairment and apoptosis of neurons in rats intrahippocampally injected with β-amyloid peptides [107], and anxiety and stress are reduced in mice in the elevated-plus maze test due to an increase in the amounts of fast nerve growth factor receptor (NGFR) [36]. Additionally, the oil reduced maternal separation anxiety in rats and decreased the expression of cytokines (IL-6 and Ccl2) in their hippocampus [108]. Alpha-pinene is responsible for the oil’s anxiolytic-like effect. It was shown that its irregular distribution in different brain areas of the *Chamaecyparis obtusa* essential oil-treated rats is associated with their emotional behavior. High concentrations of α-pinene in various brain regions outside the hippocampus and striatum were associated with an increase in locomotor activity in rats [109]. An interesting study on the influence of *Chamaecyparis obtusa* on the central nervous system was performed on a group of students. It was shown that touching a wooden board made from *Chamaecyparis obtusa* with the soles of the feet or the right hand causes a decrease in the oxy-hemoglobin (oxy-Hb) concentrations in the left and right prefrontal cortices (which is linked to a decreased activity of the prefrontal cortex) and an increase in parasympathetic activity (heart rate variability (HRV) and heart rate were used as indicators). Touching the wood with the soles of the feet also reduced the activity of the sympathetic nervous system, presenting subjective feelings to the study group. The experiment showed that the act of touching Hinoki wood can cause a physiological reaction in the body [110,111]. A similar effect was also achieved after olfactory stimulation with *Chamaecyparis obtusa* leaf oil [112].

### 3.11. Anti-Insect Activity and Plant Diseases

In addition to medical applications, *Chamaecyparis obtusa* can also be used in other areas of everyday life or the economy. For example, Hinoki bark and its extracts can be used to inhibit some tomato root diseases, especially those caused by *Fusarium oxysporum* radicis-lycopersici or *Pseudomonas solanacearum* [113]. In addition, Hinoki may have broad anti-insect applications, which might be used to produce a “human-friendly” repellent for insects. Studies indicating such use of *Chamaecyparis obtusa* are included in Table 1. Moreover, the use of wood-wool from *Chamaecyparis obtusa* to make tatami mats (used to cover the floor) has been shown to reduce the activity of house dust mites (*Dermatophagoides pteronyssinus*) for about one year [114].

### 3.12. Other Applications

In addition to the applications presented above, new uses for *Chamaecyparis obtusa* are being explored. One of them may be a therapy for dry eye disease. Indeed, the *Chamaecyparis obtusa* leaf extract has been shown to increase the concentration of antioxidant proteins in human corneal epithelial (HCE) cells. Moreover, in both mice and patients, improvements in clinical parameters were observed following treatment with the extract or a mask containing the extract, respectively [119]. Another indication may be the prevention or treatment of atherosclerosis and restenosis, as phytoncides isolated from *Chamaecyparis obtusa* reduce proliferation and migration in rat aortic smooth muscle cells (RAoSMCs) by inhibiting the phosphorylation of Akt and ERK proteins [120]. On the other hand, biological properties of *Chamaecyparis obtusa* are not necessarily limited to pharmacotherapy. For example, the antimicrobial properties can be used to produce fabrics used for making bedding or hospital scrubs. The effectiveness of such fabrics dyed with *Chamaecyparis obtusa* extract against methicillin-resistant *S. aureus*, *K. pneumoniae* and *S. aureus* has already been proven [121].

## 4. Conclusions and Future Perspectives

Plant secondary metabolites play a key role in human life and are used in many industries. Although these compounds are not necessary for the basic growth and development of the plant, they often perform very important functions in interactions with the environment. The extremely rich world of plants offers humans many valuable compounds with very wide biological properties, such as antimicrobial, antioxidant, anticancer, antidiabetic, antiasthmatic, anti-inflammatory, and antiallergic properties, or even safe compounds used in plant protection. This effect is demonstrated by *Chamaecyparis obtusa*, a plant that has been highly valued in Japanese culture for centuries. It seems to be a plant with great application possibilities, especially as a natural anti-insect agent or natural plant protection agent. It was used to build temples, shrines and traditional teahouses. This wood is often considered prestigious and used to produce high-quality wooden products. The confirmed biological properties of this plant allow us to assume that in the future, with the increasingly rapid development of in vitro plant culture techniques, there will be a radical increase in the production of this plant or in vitro cultures obtained from it in order to secure an appropriate amount of material as a starting point for the isolation of valuable phytochemicals. In addition to their wide use in biomedicine, these compounds may occupy an important place among biological plant protection products in the future. The best known in this respect, hinokitiol, has a number of properties which, with the development of new delivery methods (encapsulation in nanostructures, increased stability and prolonged release), may become extremely competitive with currently used synthetic agents, significantly reducing the negative impact on the natural environment. Additionally, dynamically developing and constantly improved metabolic engineering tools will certainly allow for the enhancement of the natural content of selected valuable secondary metabolites, which, combined with large-scale breeding, may allow the development of a very efficient plant system with industrial applications, significantly enriching the arsenal of currently used plants that are valuable from many points of view.

## Figures and Tables

**Table 1:** Use of *Chamaecyparis obtusa* in the control of insects and plant diseases.

Potential Application	Insect	Oil/Extract	Main/Important/Basic Compounds	Ref
Pest deterrent in the storage of products	*Callosobruchus chinensis* (L.), *Sitophilus oryzae* (L.)	Essential oil	bornyl acetate, (+)-limonene, myrcene, α-phellandrene, α-pinene, sabinene and terpinolene	[38]
	*Spodoptera litura* (Fabricius)	Cones extract	trans-communic acid, trans-communol, 12-hydroxy-6,7-seco-abieta-8,11, 13-triene-6,7-dial, chamaecydin, ferruginol	[26]
Mosquito repellent	Larvae of *Aedes aegypti* (L.) *Ochlerotatus togoi* (Theobald), *Culex pipiens pallens* (Coquillett)	Leaf methanol extract	β–thujaplicin	[115]
	Larvae of *Aedes aegypti* (L.) and *Culex pipiens pallens* (Coquillett)	Essential oil	-	[116]
Fly repellent	*Drosophila melanogaster* (Meigen), *Musca domestica* (L.)	Essential oil	α-terpinolene (+)-3-carene, α-pinene, sabinene, and γ-terpinen	[39]
Antithermal agent	*Reticulitermes speratus* (Kolbe)	Branch and trunk heartwood extract	hinokiresinol, germacra-1-(10), α-cadinol, t-cadinol, 5-dien-4b-ol and hinokinin	[117]
Acaricide agent	*Dermatophagoides farinae*, *Dermatophagoides* *pteronyssinus*	Leaf methanol and hexane extract	β-thujaplicin	[118]

## Data Availability

No data were used for the research described in the article.

## Abbreviations

ABTS	2,2′-azino-bis(3-ethylbenzothiazoline-6-sulfonic acid)
AD	atopic dermatitis
ALP	alkaline phosphatase
ATP	adenosine 5′-triphosphate
BALF	bronchoalveolar lavage fluid
CNS	central nervous system
COEO	Chamaecyparis obtusa essential oil
COX-2	cyclooxygenase-2
CREB	cAMP response element-binding protein
DNCB	2,4-dinitrochlorobenzene
DPPH	2,2-diphenyl-1-picrylhydrazyl
EMT	epithelial-mesenchymal transition
ERK	extracellular signal-regulated kinase
GR	glutathione reductase
HOMA-IR	homeostatic model assessment—insulin resistance
HRV	heart rate variability
HSV-1	* Herpes simplex virus 1 *
IGF-1	insulin-like growth factor 1
JAK/STAT	Janus kinases/signal transducer and activator of transcription proteins signaling pathway
JNK	c-Jun N-terminal kinase
KGF	keratinocyte growth factor
LFCO	Lactobacillus-fermented *Chamaecyparis obtusa*
MBC	minimum bactericidal concentration
MIC	minimum inhibitory concentration
MITF	microphthalmia-associated transcription factor
MRSA	methicillin-resistant *Staphylococcus aureus*
NGFR	nerve growth factor receptor
NO	nitric oxide
NSAID	non-steroidal anti-inflammatory drug
PARP	poly (ADP-ribose) polymerase
ROS	reactive oxygen species
SOD	superoxide dismutase
STAT3	signal transducer and activator of transcription 3
TGFβ1	transforming growth factor β1
TNFα	transforming growth factor α
TRP1 and TRP2	tyrosinase-related proteins
TTO	tea tree oil
VEGF	vascular endothelial growth factor
VRE	vancomycin-resistant *enterococci*
WHO	World Health Organization
α-MSH	alpha-melanocyte-stimulating hormone
γ-GT	γ-glutamyl transpeptidase
